# Comprehensive genetic analysis of pediatric germ cell tumors identifies potential drug targets

**DOI:** 10.1038/s42003-020-01267-8

**Published:** 2020-09-30

**Authors:** Yasuo Kubota, Masafumi Seki, Tomoko Kawai, Tomoya Isobe, Misa Yoshida, Masahiro Sekiguchi, Shunsuke Kimura, Kentaro Watanabe, Aiko Sato-Otsubo, Kenichi Yoshida, Hiromichi Suzuki, Keisuke Kataoka, Yoichi Fujii, Yuichi Shiraishi, Kenichi Chiba, Hiroko Tanaka, Mitsuteru Hiwatari, Akira Oka, Yasuhide Hayashi, Satoru Miyano, Seishi Ogawa, Kenichiro Hata, Yukichi Tanaka, Junko Takita

**Affiliations:** 1 Department of Pediatrics, Graduate School of Medicine, The University of Tokyo, Tokyo, Japan; 2 Department of Maternal-Fetal Biology, National Research Institute for Child Health and Development, Tokyo, Japan; 3 Clinical Research Institute and Department of Pathology, Kanagawa Children’s Medical Center, Yokohama, Japan; 4 Department of Pediatrics, Hiroshima University Graduate School of Biomedical Sciences, Hiroshima, Japan; 5 Department of Pathology and Tumor Biology, Graduate School of Medicine, Kyoto University, Kyoto, Japan; 6 Section of Genome Analysis Platform, Center for Cancer Genomic and Advanced Therapeutics, National Cancer Center, Tokyo, Japan; 7 Laboratory of DNA Information Analysis, Human Genome Center, Institute of Medical Science, The University of Tokyo, Tokyo, Japan; 8 Department of Cell Therapy and Transplantation Medicine, The University of Tokyo Hospital, Tokyo, Japan; 9 Institute of Physiology and Medicine, Jobu University, Takasaki, Japan; 10 Department of Medicine, Center for Hematology and Regenerative Medicine, Karolinska Institute, Stockholm, Sweden; 11 Institute for the Advanced Study of Human Biology (WPI ASHBi), Kyoto University, Kyoto, Japan; 12 Department of Pediatrics, Graduate School of Medicine, Kyoto University, Kyoto, Japan

**Keywords:** Cancer genomics, Germ cell tumours, Paediatric cancer

## Abstract

To elucidate the molecular pathogenesis of pediatric germ cell tumors (GCTs), we performed DNA methylation array analysis, whole transcriptome sequencing, targeted capture sequencing, and single-nucleotide polymorphism array analysis using 51 GCT samples (25 female, 26 male), including 6 germinomas, 2 embryonal carcinomas, 4 immature teratomas, 3 mature teratomas, 30 yolk sac tumors, and 6 mixed germ cell tumors. Among the 51 samples, 11 were from infants, 23 were from young children, and 17 were from those aged ≥10 years. Sixteen of the 51 samples developed in the extragonadal regions. Germinomas showed upregulation of pluripotent genes and global hypomethylation. Pluripotent genes were also highly expressed in embryonal carcinomas. These genes may play essential roles in embryonal carcinomas given that their binding sites are hypomethylated. Yolk sac tumors exhibited overexpression of endodermal genes, such as *GATA6* and *FOXA2*, the binding sites of which were hypomethylated. Interestingly, infant yolk sac tumors had different DNA methylation patterns from those observed in older children. Teratomas had higher expression of ectodermal genes, suggesting a tridermal nature. Based on our results, we suggest that KIT, TNFRSF8, and ERBB4 may be suitable targets for the treatment of germinoma, embryonal carcinomas, and yolk sac tumors, respectively.

## Abstract (web summary)

Yasuo Kubota et al. report a multi-omic analysis of pediatric germ cell tumors from 51 patients ranging in age from 2 months to 19 years. They identify unique methylation, expression, and mutational patterns for each of the main subtypes and propose potential target genes for treatments against the three main subtypes.

## Introduction

Germ cell tumors (GCTs) are relatively rare in pediatric patients, and the incidence rates are 5.3 and 4.4 per million in girls and boys, respectively^1^. GCTs usually exhibit a bimodal age distribution with two peaks in infancy and young adolescence^2^. The survival rate of patients is excellent (>90%)^1^. However, in some cases, GCTs are refractory to treatment, including intensive chemotherapy^3^. Therefore, a novel treatment strategy should be established for chemotherapy-resistant GCTs. Additionally, a new approach of treatment may even be required for chemotherapy-sensitive GCTs. It has been found that survivors of pediatric cancer suffer from late complications induced by anticancer cytotoxic agents^4^. Therefore, we should elucidate the molecular pathogenesis of GCTs and develop novel therapies based on it.

GCTs develop from primordial germ cells (PGCs). PGCs develop in the yolk sacs of embryos during the embryonic period and migrate to gonads through the midline of the body^5^. As PGCs migrate to the gonads, they undergo DNA methylation erasure^6^. In this process, the KIT and CXCR4 pathways are associated with proliferation and migration, respectively^7^. After migration, PGCs reestablish their DNA methylation and differentiate into sperms or eggs depending on the sex of the individual^8^. Migrating PGCs are chromosomally unstable due to DNA hypomethylation and are prone to developing copy number abnormalities or mutations^9^. Although such aberrated PGCs are eliminated via apoptosis, a minority of aberrated PGCs survive and give rise to GCTs^10^. GCTs develop in the gonads and extragonads^11^, such as the central nervous system (CNS), mediastinum, or sacrococcygeal regions, which are thought to be the migration routes of PGCs. While GCTs include several subtypes, such as seminoma, yolk sac tumors (YSTs), or embryonic carcinomas (ECs), they are dependent on the differential stages of PGCs^10^.

In adults, the most common subtype of testicular GCTs is an immature type of GCT called seminoma^12^. Seminomas frequently exhibit an isochromosome of the short arm of chromosome 12 (isochromosome 12p) as well as mutations in *KIT* and *RAS*^13^. Because seminomas comprise neoplastic counterparts of PGCs, they exhibit global DNA hypomethylation^14^. Immature types of GCTs in the ovary and CNS are known as dysgerminomas and germinomas, respectively. In this study, dysgerminomas, seminomas, and CNS germinomas were all classified as germinomas. Because they also commonly retain global DNA hypomethylation, isochromosome 12p, and *KIT* or *RAS* mutations^15–18^, these tumors could have similar biological characteristics to seminomas. In fact, as isochromosome 12p is detected in adult GCTs regardless of their subtypes, it is considered to be one of the critical factors responsible for the development of GCTs. Additionally, DNA methylation aberrations could be associated with the development of GCTs owing to the dynamics of DNA methylation. In YSTs, several tumor-suppressor genes (such as *RASSF1* or *APC*) are hypermethylated^14,19^. These reports suggest that DNA methylation aberrations, in addition to copy number abnormalities and mutations, contribute to the development of GCTs. Several studies have investigated genomic abnormalities in pediatric GCTs^20,21^; however, a comprehensive genomic analysis of pediatric GCTs has not been performed. Therefore, using 51 pediatric GCT samples, we performed a comprehensive genetic/epigenetic analysis including DNA methylation array, whole-transcriptome sequencing, single-nucleotide polymorphism (SNP) array, and targeted capture sequencing to obtain a better understanding of the molecular pathogenesis of pediatric GCTs.

Our comprehensive analyses profiled genetic and epigenetic characteristics in each subtype of pediatric GCTs. Germinomas showed upregulation of pluripotent genes and global hypomethylation. Pluripotent genes were also highly expressed in ECs. These genes may play essential roles in ECs given that their binding sites are hypomethylated. YSTs exhibited overexpression of endodermal genes, such as *GATA6* and *FOXA2*, the binding sites of which were hypomethylated. Interestingly, infant YSTs had different DNA methylation patterns from those observed in older children. Teratomas had higher expression of ectodermal genes, suggesting a tridermal nature. Based on our results, we suggest that KIT, TNFRSF8, and ERBB4 may be suitable targets for the treatment of germinoma, ECs, and YSTs, respectively.

## Results

A total of 51 patients with pediatric GCT (Table 1 and Supplementary Data 1) were enrolled in this retrospective study. All samples were collected from the University of Tokyo Hospital and associated hospitals. All patients received carboplatin-based chemotherapy at each hospital. Our cohort included 6 patients with germinomas, 2 with ECs, 4 with immature teratomas, 3 with mature teratomas, 30 with YSTs, and 6 with mixed germ cell tumors (MGCTs). Among these 51 samples, 16 developed in the extragonadal regions. The male-to-female ratio of our cohort was almost similar (26 males and 25 females). Among the 51 samples, 11 were from infants, 23 were from those aged 1 to <10 years, and 17 were from those aged ≥10 years. The ages ranged from 2 months to 19 years, with a median age of 2 years. The overall prognosis was excellent, with three recurrences and two deaths reported.

**Table 1:** Clinical characteristics of the patients.

	Number	Proportion
Sex		
Male	26	(0.51)
Female	25	(0.49)
Age of onset		
Under 1 year	11	(0.22)
1–9 years	23	(0.45)
Over 10 years	17	(0.33)
Subtype		
Germinoma	6	(0.12)
Embryonal carcinoma	2	(0.04)
Immature teratoma	4	(0.08)
Mature teratoma	3	(0.06)
Yolk sac tumor	30	(0.59)
Mixed germ cell tumor	6	(0.12)
Primary lesions		
Testis	19	(0.37)
Ovary	16	(0.31)
Mediastinum	7	(0.14)
Sacrococcygeal	6	(0.12)
Retroperitoneal	3	(0.06)
Outcome		
Alive^a^	49	(0.96)
Dead^b^	2	(0.04)

^a^Includes one relapsed patient.

^b^Includes two relapsed patients.

### DNA methylation analysis

**Fig. 1:**
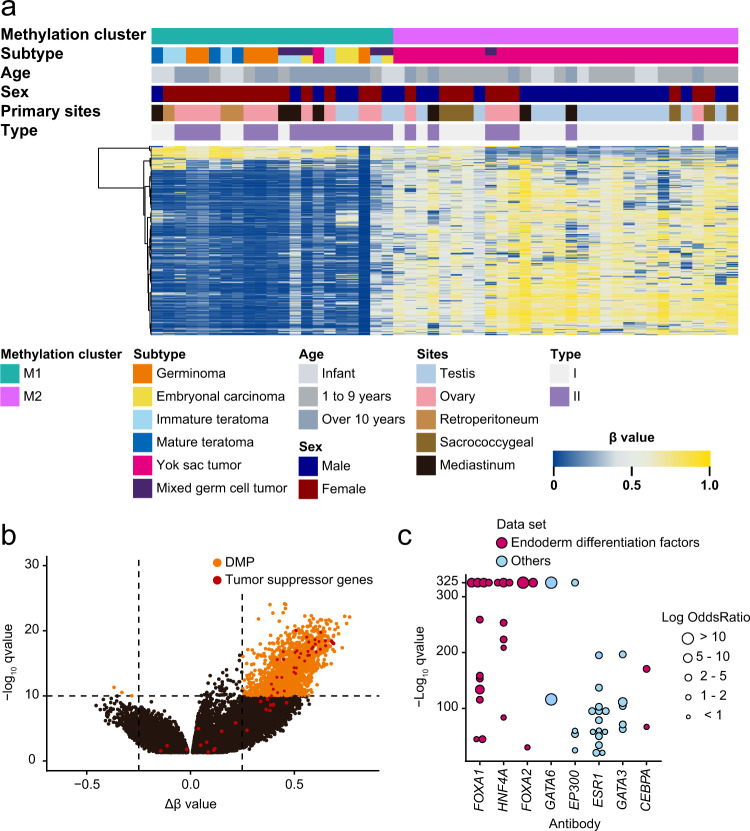
DNA methylation analysis. **a** DNA methylation clustering in 51 GCT samples on unsupervised consensus clustering. Consensus clustering revealed that GCT samples were divided into two clusters: M1 and M2. While M1 included several subtypes except YSTs, M2 included almost all YSTs. MGCTs were clustered depending on the main subtypes. DNA methylation clusters, subtypes, age at onset, sex, primary developing sites, and types are shown by colors as indicated. MGCTs are also annotated by their main subtypes. **b** Volcano plot comparing significant delta–beta values between M1 and M2 clusters. Differentially methylated probes showing a delta–beta value >0.25 or <−0.25 and −log_10_ (*q*-value) >10 are colored in orange. Among these significant probes, the probes of the promoter regions of tumor-suppressor genes are colored in red. *q*-values are calculated using the Wilcoxon rank-sum test after adjusting by Benjamini–Hochberg correction. **c** The result of locus overlap analysis between M1 and M2 clusters. Colored dots represent ChIP-seq experiments for transcription factors. Dot size denotes the log-odds ratio. Among these dots, the dots of endoderm differentiation factors are colored in red. The binding sites of *FOXA1*, *HNF4A*, *FOXA2*, *GATA6*, *EP300*, *ESR1*, *GATA3*, and *CEBPA* were hypomethylated in the M2 (YST) cluster.

The density plot of each subtype revealed that germinomas were hypomethylated versus other subtypes (Supplementary Fig. 1). Consensus clustering of our cohort revealed that our samples were divided into two clusters: M1 and M2 (Fig. 1a and Supplementary Data 2). While the M1 cluster included several subtypes, the M2 cluster included almost all YSTs. MGCTs were clustered depending on the main subtypes. The two clusters were stable, and YSTs occupied two-third of our cohort. Actually, clustering based on the same probe sets (Supplementary Data 1) separated the open data^22^ of GCTs into YSTs and other subtypes (Supplementary Fig. 2). In a previous report^14^, differentially methylated regions (DMRs) between M1 and M2 showed that the tumor-suppressor genes, such as *RASSF1*, *RUNX3*, *HIC1*, or *APC*, were hypermethylated in M2, namely, in YSTs (Fig. 1b and Supplementary Data 3). Locus overlap analysis (LOLA) revealed that the binding sites of endodermal differential factors, particularly those of hepatic factors (e.g., *FOXA2* and *HNF4A*), were hypomethylated (Fig. 1c and Supplementary Data 4). In addition, the binding sites of *GATA6* were hypomethylated in M2. The open data of adult YSTs also showed hypomethylation of the binding sites of *GATA6*, *FOXA2*, and *HNF4A*^23^ (Supplementary Fig. 3).

**Fig. 2:**
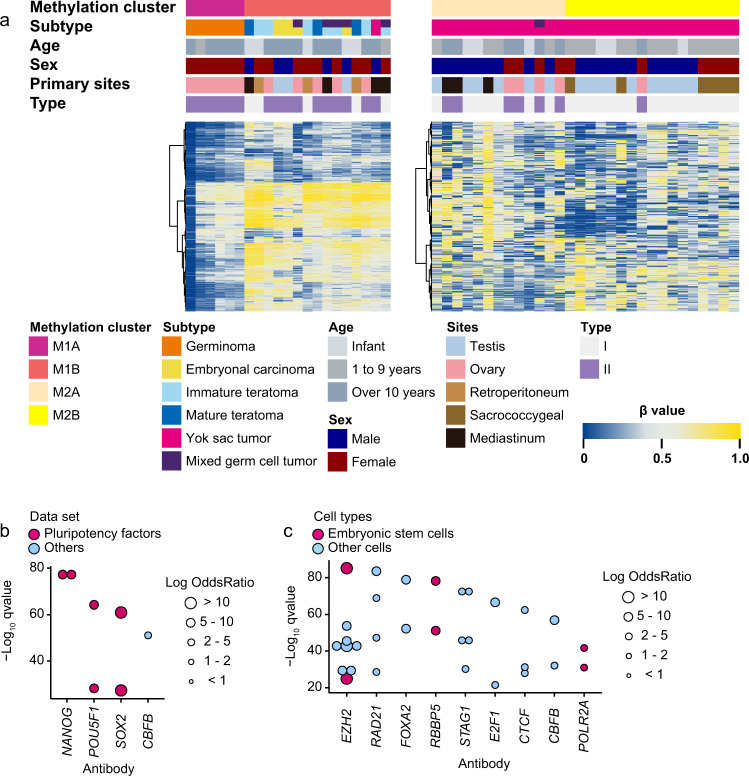
DNA methylation analysis based on two-step unsupervised consensus clustering. **a** DNA methylation clustering in 21 samples of the M1 cluster (left panel) and in 30 samples of the M2 cluster (right panel) on unsupervised consensus clustering. A second consensus clustering revealed that M1 and M2 are further divided into two clusters. M1 cluster are separated into germinomas (M1A) and ECs and teratomas (M1B) clusters. M2 clusters are divided into adult-type (M2A) and infant-type (M2B) YSTs. DNA methylation clusters, subtypes, age at onset, sex, primary developing sites, and types are shown by colors as indicated. **b** Result of locus overlap analysis between embryonal carcinomas and other subtypes. Colored dots represent ChIP-seq experiments for transcription factors. Dot size denotes the log-odds ratio. Among these dots, those of pluripotency factors are colored in red. The binding sites of *NANOG*, *POU5F1*, *SOX2*, and *CBFB* were hypomethylated in embryonal carcinomas. **c** Result of locus overlap analysis between adult-type (M2A) and infant-type (M2B) YSTs. Colored dots represent ChIP-seq experiments for transcription factors. Dot size denotes the log-odds ratio. Among these dots, those of embryonic stem cells are colored in red. Infant-type YST has partially common methylation pattern with ES cells.

We performed a second consensus clustering of the M1 samples to determine the methylation profiles of the GCT subtypes in the M1 cluster as well as a second consensus clustering of the M2 samples so as to further classify the YST samples in the M2 cluster. The second consensus clustering of the M1 and M2 samples revealed that the M1 samples were divided into germinomas (M1A cluster) and other subtypes (M1B cluster), whereas the M2 samples were divided into two YSTs clusters: M2A and M2B (Fig. 2a and Supplementary Data 5 and 6). Consensus clustering revealed that methylation profiles depended on the histological subtypes, consistent with the findings of previous studies^20,24^. Thus comparing each subtype with other subtypes led to the detection of DMRs in each subtype. However, characteristic DMRs were not detected owing to hypomethylation of almost all genes of germinomas (M1A). While M1B included several subtypes, such as ECs, immature teratomas, and mature teratomas, DMRs were determined by each subtype. In ECs, the binding sites of pluripotency factors, such as *NANOG*, *POU5F1*, and *SOX2*, were hypomethylated (Fig. 2b and Supplementary Data 7). The open data of adult ECs also showed hypomethylation of the binding sites of *NANOG*, *POU5F1*, and *SOX2*^23^ (Supplementary Fig. 4). We did not identify characteristic DMRs of teratomas compared with other subtypes and DMRs between mature and immature teratomas^24^. The M2 cluster was divided into two clusters: M2A and M2B. Comparison of M2A with M2B samples revealed slight hypermethylation in the latter (Supplementary Fig. 5). LOLA revealed that M2B samples had hypomethylation of the binding sites of *EZH2* or *RBBP5* in ES cells (Fig. 2c and Supplementary Data 8). This result suggests that M2B samples possess unique methylation patterns different from those observed in M2A samples.

### Expression analysis

**Fig. 3:**
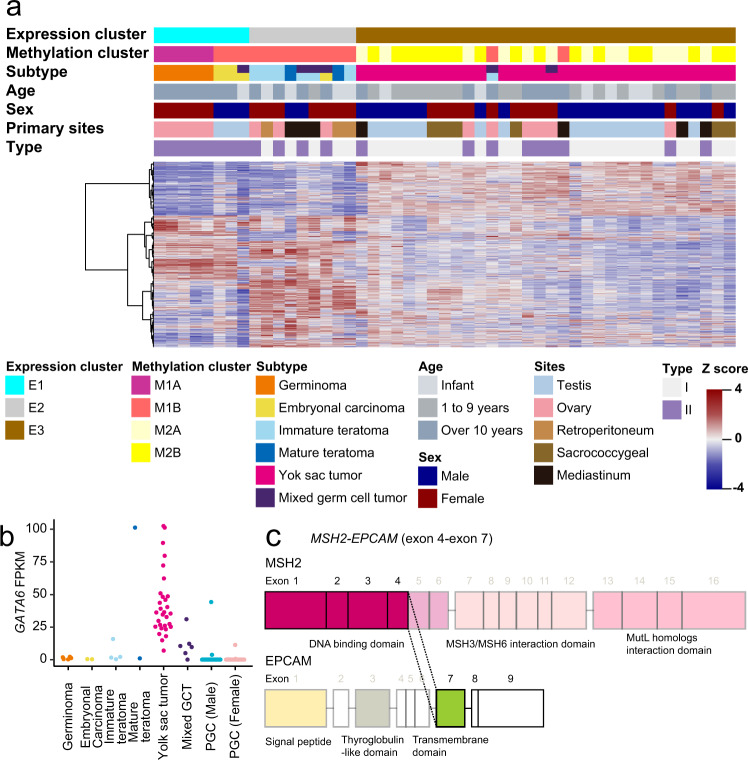
Gene expression analysis. **a** Expression clustering in 49 GCT samples on unsupervised consensus clustering. Consensus clustering revealed that GCT samples are divided into three clusters: E1, E2, and E3. E1 included all germinomas and embryonal carcinomas. E2 included all teratomas. E3 included all yolk sac tumors. As with DNA methylation clustering, expression clustering correlated with histological subtypes. MGCTs were clustered dependent on the main subtypes. DNA methylation clusters, subtypes, age at onset, sex, primary developing sites, and types are shown by colors as indicated. **b** FPKM values of *GATA6* in subtypes of GCTs and PGCs of the open data set. *GATA6* is significantly highly expressed in YSTs than in PGCs. **c** Schematic representation of the *MSH2–EPCAM* fusion gene. The exon 4 of *MSH2* is connected with the exon 7 of *EPCAM*. Because *MSH2* loses MSH3/MSH6 interaction and MutL homologs interaction domains, *MSH2* loses its function.

Expression analysis showed that GCTs were divided into three clusters (Fig. 3a and Supplementary Data 9). The result of clustering based on expression also correlated with the histological subtypes. E1 included germinoma and EC samples, E2 included all teratoma samples, and E3 included all YSTs.

E1 was characterized by a higher expression of pluripotency factors, including *POU5F1* or *NANOG* (Supplementary Fig. 6a), which was concordant with previous reports^25,26^. Expression analysis revealed that the EC samples were clustered with germinoma samples because they also exhibited higher expression of pluripotency factors. Notably, in DNA methylation clustering, EC samples were clustered with teratomas. Additionally, the MGCT sample including the EC component was also clustered into E1. Comparing ECs with germinomas in cluster E1, ECs overexpressed *SOX2*, *GDF3*, and *TNFRSF8*^27^ (Supplementary Fig. 6b). In addition, *DPPA3* and *KIT* were highly expressed in ECs^28^ and germinomas, respectively, although the difference was not significant due to the small sample size.

Mature teratomas, immature teratomas, and MGCTs, which included the teratoma component, were clustered into E2. Analysis of differential expression genes revealed that ectodermal genes, particularly the neuroectodermal genes, were highly expressed in teratomas (Supplementary Fig. 6c). Tumorigenic genes associated with the development of teratoma were not detected in genes exhibiting higher expression in E2. Comparison of immature teratomas with mature teratomas revealed that the former had higher *SOX2* expression, suggesting their immaturity.

E3 included YSTs and MGCTs with the YST component; therefore, the expression characteristics of MGCT samples in cluster E3 were similar to those of YST. YST samples overexpressed endodermal induction genes, such as *FOXA2* or *HNF4A* and *GATA6* (Supplementary Fig. 6d)^21^. Actually, because the expression of *GATA6* was not detected in the open data of PGC in fetuses^29^, the expression of *GATA6* would be specific for YSTs (Fig. 3b). Pathway analysis revealed activation of the transforming growth factor-β signaling pathway in YSTs, similar to the findings of a previous report^21^, which was concordant with the hypermethylation of *RUNX3* and *APC* (Supplementary Data 10). Additionally, *ERBB4* was highly expressed in YSTs regardless of the regions of development or the age at onset. Analysis of differentially expressed genes between the M2A and M2B clusters in DNA methylation did not detect specific genes associated with pathogenicity. RNAseq also revealed that one YST sample carried the *EPCAM–MSH6* fusion gene (Fig. 3c).

### Copy number and mutational analyses

**Fig. 4:**
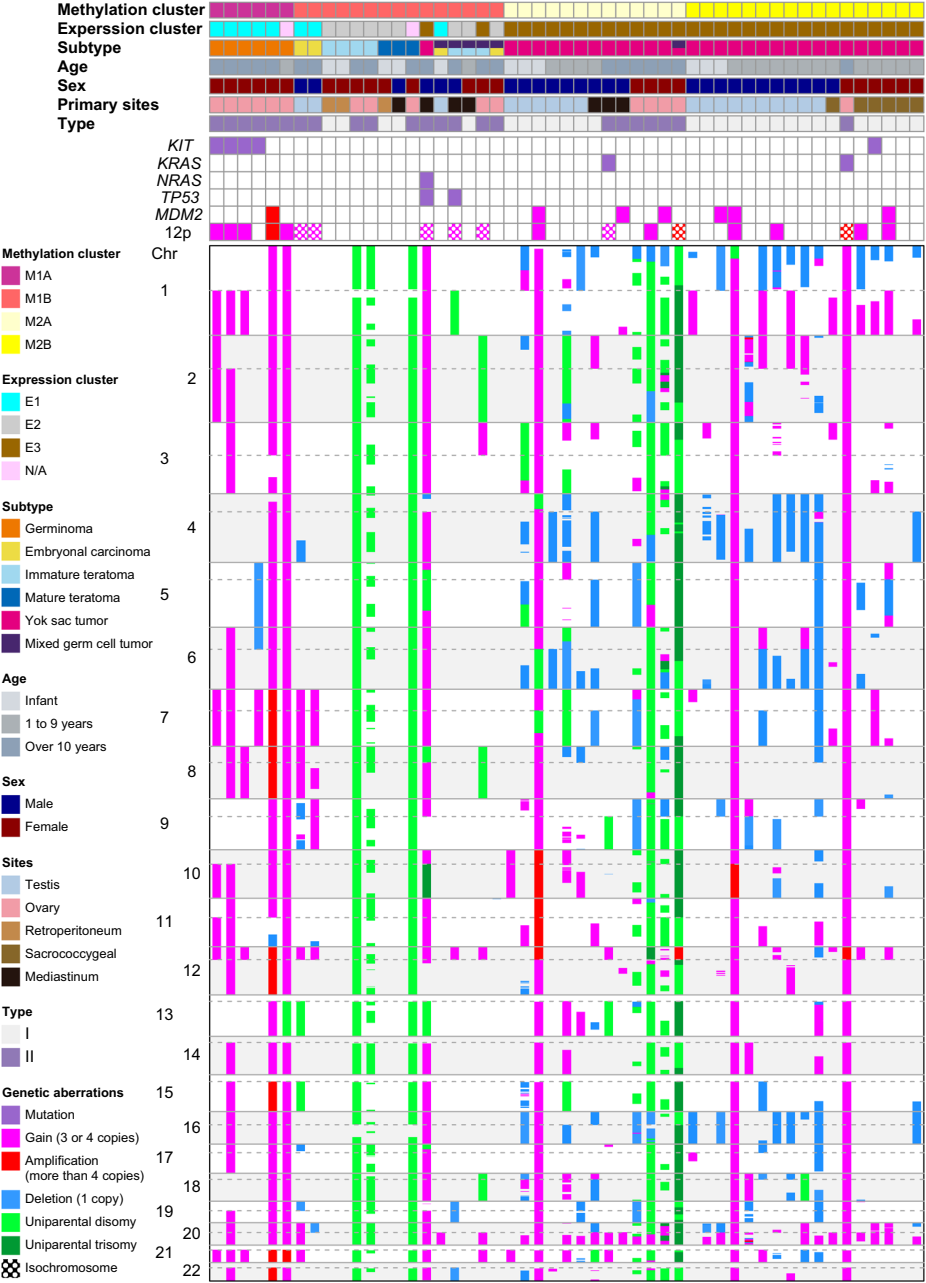
Significant somatic mutations, focal copy number alterations, and chromosomal changes in 51 GCT samples. DNA methylation clusters, subtypes, age at onset, sex, primary developing sites, and types are shown by colors as indicated. Germinomas show chromosomal gains in nearly all chromosomes or *KIT* mutations. Two ECs have isochromosome 12p and gains of chromosome 7. Teratomas do not reveal specific mutations or copy number changes except global UPD in ovarian teratomas. YSTs exhibit gains of chromosome 1q, 12p, and 20q; losses of 1p, 4p, 6p, and 16; and partial UPD. Some YST samples possess *KIT* mutation; *KRAS* mutations; and *NRAS* mutation, *TP53* mutations, or *MDM2* amplifications.

Copy number and mutational analyses revealed that each subtype had characteristic copy number alterations (Fig. 4) and mutations (Supplementary Data 11). In general, gaining of the long arms of chromosome 20 was the most frequently detected copy number alterations in 29 of the 51 GCT samples regardless of the histological subtypes, except teratomas. In addition to 20q, gaining of the short arms of chromosome 12 was also frequently detected in 20 of the 51 GCT samples. Among these 20 samples, 8 samples carried isochromosome 12p (Supplementary Fig. 7). All samples with isochromosome 12p belonged to children aged >10 years.

While two germinoma samples showed chromosomal gains in nearly all chromosomes, other germinoma samples had fewer copy number alterations. Gains in chromosomes 1q, 7, 8, 10q, 12p, 20q, and 21q were detected in five of six germinoma samples. In our cohort, none of the germinoma sample possessed isochromosome 12p, which is often detected in adult germinomas^30^. Two germinoma samples had more than four copies of chromosome 12p, and these two germinoma samples did not have *KIT* mutations. In contrast, all germinoma samples with three or fewer copies of chromosome 12p carried activating mutation in *KIT*, which is one of the most frequently mutated genes detected in adult germinomas^31^. Two germinoma samples with more than four copies of chromosome 12p also gained the long arms of chromosome 4, which included *KIT*.

Although none of the EC samples carried mutations, they recurrently showed isochromosome 12p as well as gains of chromosome 7 and 8q. Expression analysis revealed that *GDF3* and genes were highly expressed in ECs and were located in chromosome 12p^23^. Overexpression of these genes might be associated with gains of chromosome 12p.

Although teratomas did not exhibit characteristic copy number alterations or mutations, ovarian teratomas had global uniparental disomies (UPDs; Supplementary Fig. 8). Global UPD in ovarian teratomas was frequently found in adult ovarian teratomas^32^.

In 30 YST samples, gains of chromosome 1q (14 samples), 12p (nine samples), and 20q (23 samples); losses of 1p (17 samples), 4q (13 samples), 6q (13 samples), and 16 (13 samples)^33^; and partial UPD or uniparental trisomies (UPT) (11 samples) were frequently observed. There were several differences between the M2A and M2B cluster samples according to DNA methylation analysis. While the M2A cluster samples had more partial UPD or UPT, the M2B cluster samples had more 1q gains. Isochromosome 12p in YST samples was detected only in older children. Furthermore, two of the three YST samples with chromosomal gains in nearly all chromosomes developed in older children. While UPD or UPT was detected in the gonadal or extragonadal samples of male YST samples, only UPD was detected in the gonadal samples of female YST samples. In particular, ovarian YST samples showed global UPD similar to ovarian teratomas (Supplementary Fig. 9). In YST samples, *KIT*, *KRAS*, and *NRAS* mutations were detected in one, two, and one sample, respectively. These samples belonged to children aged >10 years. MGCT samples correlated with the mixture of several subtypes and possessed copy number alterations characteristic of each subtype.

Nonsynonymous *TP53* mutations were detected in one YST (NM_001126114: p.C141Y) and one MGCT (NM_001126114: p.E11Q) sample belonging to children aged >10 years and developed in the mediastinum. The gains of *MDM2* were detected in six YST samples, which highly expressed *MDM2* versus other samples (Supplementary Fig. 10).

## Discussion

**Fig. 5:**
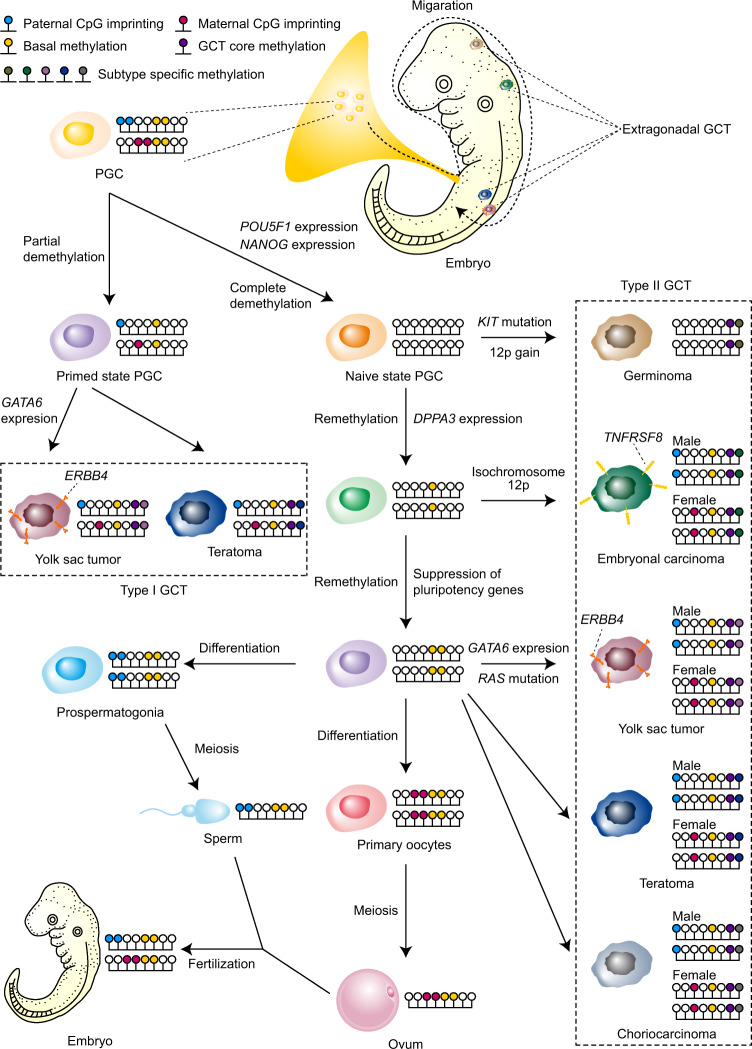
Schematic representation of developing GCTs. PGCs develop in the yolk sacs of embryos in the embryonic period and migrate to gonads through the midline of the body. As PGCs migrate to gonads, they undergo DNA methylation erasure, including imprinting regions and basal methylation. With GCT core methylation and subtype-specific methylation, infant GCTs and type I GCTs develop from primed PGCs, which are partially demethylated PGCs. After migration and complete DNA demethylation, PGCs reestablish their DNA methylation and express pluripotent genes. PGCs expressing pluripotency genes gain additional genomic abnormalities, such as *KIT* mutation or 12p gain, and develop into germinomas. PGCs expressing pluripotent genes further reestablish DNA methylation and express *DPPA3*. PGCs expressing *DPPA3* sometimes gain isochromosome 12p and develop into ECs. PGCs expressing *DPPA3* further reestablish DNA methylation and differentiate into sperms or eggs dependent on each sex. On the way of differentiation, PGCs gaining additional genomic abnormalities develop into YSTs or teratomas. As type II EC, YST, and teratoma possessed sex-specific imprinting in the analysis of imprinting regions, these tumors are shown by each sex. This figure does not include type II GCTs developed in the extragonads.

Our comprehensive analysis demonstrated that pediatric GCTs had characteristic genomic aberrations based on their developmental processes (Fig. 5).

As germinomas showed global hypomethylation and higher expression levels of pluripotency factors (e.g., *POU5F1* or *NANOG*), they could develop from immature PGCs^14,22^. Although DNA methylation and expression analyses revealed that germinomas were uniform, copy number and mutational analyses could separate germinomas into two types. The first type possessed activating *KIT* mutations and fewer copy number alterations, suggesting that the activation of the KIT pathway was essential for some germinomas. The second type exhibited chromosomal gains in nearly all chromosomes without *KIT* mutations. These two types were mutually exclusive in our cohort. However, the expression levels of *KIT* were similar between the two types of germinomas. The karyotypes of the second type were near triploid or tetraploid, and these karyotypes were reported in adult GCTs^34^. Different from the germinomas in our cohort, most adult seminomas carried isochromosome 12p^13^, and some seminomas with isochromosome 12p retained activating *KIT* mutations, suggesting that the acquisition of isochromosome 12p precedes that of *KIT* mutations. In our cohort, five of the six germinoma samples had 12p gains. Among the five samples, two had more than four copies of 12p without *KIT* mutations. Therefore, a larger number of 12p copies may be able to cause the development of germinomas similar to that caused by *KIT* mutations; however, we did not identify any other causative genes except *KIT*. In this study, the outcome of germinomas was excellent; nevertheless, some adult germinomas are refractory to chemotherapy^35^. In pediatric intracranial germinomas, *KIT*-mutated cases were linked to worse prognosis^22^. The KIT inhibitor, avapritinib, is effective for the treatment of systemic mastocytosis with *KIT* D816 mutations^36^. Therefore, avapritinib may also be a promising therapeutic option for germinomas with *KIT* mutations.

ECs exhibited more methylated regions and higher expression of *DPPA3* than germinomas; therefore, it was concluded that ECs developed from differentiated PGCs^23^. The observed overexpression of pluripotency factors and hypomethylation of their binding sites suggest that these factors play essential roles in ECs. The presence of the expression of pluripotency factors indicated the immature states of the ECs. Other cancers (e.g., renal cell carcinoma) with higher expression of pluripotency factors are linked to worse prognosis^37^. The worse prognosis of ECs in non-seminomas^38^ may also be associated with pluripotency factors. Because *TNFRSF8* was highly expressed in ECs in our cohort, brentuximab may be a promising option for the treatment of refractory ECs. While copy number abnormalities induce the expression of *TNFRSF8*^39^, other subtypes with complexed copy number abnormalities did not cause this effect. This finding suggests that *TNFRSF8* expression is characteristic to ECs. Although ECs in our cohort did not possess mutations and adult ECs rarely have driver mutations^13^, most ECs carry isochromosome 12p^23^. Therefore, the present results imply that 12p gains may be indispensable for the development of ECs because 12p includes various genes associated with cancer (e.g., *DPPA3*, *GDF3*, *KRAS*, or *CCND2*).

Owing to the suppression of pluripotency factors, other nonseminomatous subtypes would be more differentiated than ECs. The teratomas in our cohort had similar DNA methylation and expression profiles. Compared with other subtypes, teratomas had higher expression of ectodermal genes, particularly of neuroectodermal genes, because they were tridermal tumors. In contrast, copy number analysis showed that only ovarian teratomas had global UPDs, suggesting the parthenogenetic development of ovarian teratomas^40^. Because teratomas had similar genomic profiles regardless of the presence or absence of UPDs, imprinting genes may play limited roles in the development of teratomas.

YSTs overexpressed endodermal genes (e.g., *FOXA2* or *HNF4A*) and extraembryonic genes (e.g., *GATA6*)^41^. DNA methylation analysis revealed that the binding sites of these genes were hypomethylated; therefore, these genes would play fundamental roles in YSTs in a similar manner to pluripotency factors in ECs. *GATA6* is particularly important among these genes because it is the master regulator of the differentiation of extraembryonic tissues and induces the differentiation of extraembryonic tissues, such as yolk sacs^42^ during the embryonic period. Pathway analysis showed activation of the transforming growth factor-β pathway in YSTs, which is associated with the overexpression of *GATA6* and *FOXA2* and the hypermethylation of tumor-suppressor genes (e.g., *RASSF1*, *RUNX3*, *HIC1*, or *APC*). Additionally, *ERBB4* was also upregulated in YSTs. Because ERBB4 inhibition is effective for the treatment of breast cancer^43^, it may also be a promising therapeutic option against YSTs. The *EPCAM–MSH6* gene fusion detected in one YST sample is one of the causative genes of Lynch syndrome, which may be a risk factor of the development of adult testicular GCTs^44^.

DNA methylation analysis divided the YST samples into two clusters: M2A and M2B; however, the expression profiles of these two types were similar. While the M2A cluster included almost all type II YST and mediastinal samples, the M2B cluster included all type I YST and sacrococcygeal samples. Generally, type I and type II GCTs are pediatric and adult types, respectively. In addition, all sacrococcygeal GCTs were of the infant type, and mediastinal GCTs were rare in children. Thus samples from the M2A and M2B clusters could be the so-called adult and infant GCTs, respectively. While infant GCTs developed from migrating PGCs undergoing DNA methylation erasure, adult GCTs developed from PGCs reestablishing DNA methylation. These results imply differences in DNA methylation between adult and infant GCTs. Consistent with this prediction, comparison between M2A and M2B samples revealed slight hypermethylation in the latter.

Copy number status was partly different between infant- and adult-type YSTs. In addition, ovarian YSTs showed global UPD similar to ovarian teratomas. Therefore, ovarian YSTs and teratomas may have the same origin because type I MGCTs (including YSTs and teratomas) were frequently detected in the pediatric type. Actually, a previous report identified the stem cells of type I GCTs^45^.

*KIT*, *KRAS*, and *NRAS* mutations were detected in one, three, and one YST sample, respectively. Although the frequency of gene mutations was low, mutations in these genes may be additional drivers for the development of YSTs.

Based on DNA methylation analysis, one YST sample was classified in the M1 cluster. While this particular sample showed higher expression of *GATA6*, *FOXA2*, or *HNF4A* and hypermethylation of *RASSF1*, *RUNX3*, *HIC1*, or *APC*, its methylation pattern was partially common to that of germinomas or ECs. Review of the pathological specimens of this particular sample showed that this particular sample contained syncytiotrophoblastic giant cells (STGCs) but not other histological components. Because STGCs are often detected in germinomas, germinomas may be incorporated into this particular YST sample. In addition to the histological involvement of STGCs, this sample possessed a *TP53* mutation and was developed in the mediastinum of a patient aged >10 years. Such YSTs may have a unique profile. As MGCTs in older children also carries the *TP53* mutation, mediastinal GCTs in older children exhibit a higher frequency of *TP53* mutations (similar to adult GCTs^46^). Thus older children may be at a higher risk of mediastinal GCTs, similar to adults, because *TP53*-mutated GCTs are refractory to chemotherapy^46^.

Our study has some limitations. First, it was unclear whether we correctly characterized the genomic and epigenomic status of each subtype because the sample size was small, except for YSTs. In particular, the identification of the subtype refractory to convention chemotherapy remains unclear. *TP53* mutations or EC subtypes might be refractory to conventional chemotherapy as discussed above. However, considering that *TP53* mutations and EC subtypes are more common in adults, the worse prognosis of these subtypes in our cohort may be correlated with differences between pediatric and adult GCTs. Thus future larger-scale studies are needed to verify our speculations. Second, the genomic and epigenomic statuses of intracranial GCTs were also unclear as intracranial GCTs were not included in this study. However, analysis using the open data set of DNA methylation data of intracranial GCTs and testicular GCTs revealed that these GCTs had a similar DNA methylation pattern according to each subtype. Therefore, GCTs could have similar biological profiles according to each subtype regardless of the primary site. Third, although the reproducibility of our results could not be confirmed using the open data sets of GCTs, our analysis possesses a certain validity given that LOLA and clustering based on DNA methylation revealed that our cohorts and the open data set had similar results.

In conclusion, our comprehensive genomic/epigenomic analyses identified the characteristics of pediatric GCTs, and each subtype possessed unique characteristics. Although pediatric GCTs are associated with favorable prognosis, some pediatric GCTs such as *TP53* mutations or EC subtype as shown in our analysis are refractory to conventional chemotherapy. For such cases, the molecular targets detected through our comprehensive genomic/epigenomic analyses may be promising therapeutic options. Therefore, additional studies are warranted to confirm the efficacy of these molecular targeted agents in patient-derived cell lines or xenograft models and to assess their effectiveness in clinical trials.

## Methods

### Patients and materials

All samples were collected from the University of Tokyo Hospital and associated hospitals. Tumor samples were collected after obtaining written informed consent from the legal guardians of the patients according to protocols approved by the Human Genome, Gene Analysis Research Ethics Committee of the University of Tokyo, and other participating institutions. All protocols conformed to the tenets of the Declaration of Helsinki. This retrospective study enrolled 51 patients with type I (infant-type) and type II (adult-type) extracranial GCTs (Supplementary Data 1)^47^. Moreover, dysgerminomas, seminomas, and CNS germinomas were all classified as germinomas.

### Statistics

Statistical analyses were performed using the R software version 3.5.1 (https://www.R-project.org/).

### DNA methylation analysis

Comprehensive DNA methylation analysis was performed using 51 samples of GCTs with the Infinium methylationEPIC Kit (Illumina, San Diego, CA, USA) according to the manufacturer’s instructions. Quality control, signal correction, and methylation beta value calculation were performed using the Bioconductor package ChAMP version 2.10.1. Differentially methylated CpG probes were ranked according to adjusted *P* values calculated by fitting linear models. For clustering, beta-values were corrected for probe design bias using a beta-mixture quantile normalization method^48^ and then converted to *M*-values^49^. Thereafter, the R package pcaMethods 1.68.0 was used to impute incomplete *M*-values, which were subsequently converted to beta-values. Imputed beta-values were later used for further analyses. For the determination of DNA methylation profiles, the following steps were adopted to select probes for unsupervised consensus clustering. Initially, we removed probes that were designed for sequences on the X and Y chromosomes. Subsequently, we selected probes annotated as promoter-associated CpG islands according to Illumina (Illumina, San Diego, CA, USA). We then performed unsupervised consensus clustering using the Ward’s method. Cluster stability was determined via consensus clustering with 1000 iterations using the R package ConsensusClusterPlus 1.40.0. Heat maps were generated using the R package pheatmap 1.0.7 using beta-value data. Statistical significance was assessed using Wilcoxon’s rank-sum test, and values were corrected by employing the Benjamini–Hochberg method. LOLA was performed using the R package LOLA 1.14.0.

### Next-generation sequencing (NGS)

NGS was performed using the Illumina HiSeq 2000 or 2500 platform (Illumina, San Diego, CA, USA) with a standard 126-bp paired-end read protocol according to the manufacturer’s instructions.

### RNA sequencing

High-quality RNA samples were isolated from 49 GCTs (RNA integrity number equivalent >5.5) using the Agilent TapeStation (Agilent Technologies, Santa Clara, CA, USA). These samples were used to prepare libraries for RNA sequencing using the NEBNext Ultra RNA Library Prep Kit for the Illumina platform (New England BioLabs, Ispwich, MA, USA). Fusion transcripts were detected using the Genomon version 2.5.3 (https://github.com/Genomon-Project/) and were filtered by excluding fusion mapping to repetitive regions, those with fewer than seven spanning reads, those occurring out of frame, or those with junctions not located at known exon–intron boundaries. Normalized count data obtained using the variance-stabilizing transformation function of the DESeq2 R package (1.16.1) were used for clustering analysis. For this analysis, we performed unsupervised consensus clustering using the Ward’s method. Heat maps were generated using the R package pheatmap version 1.0.7 using the variance-stabilizing transformation count data. Statistical significance was assessed using the Wilcoxon’s rank-sum test, and values were corrected by employing the Benjamini–Hochberg method.

### Copy number analysis

DNA samples were processed via SNP array analysis using the Affymetrix GeneChip 250K Nsp or CytoScan HD (Affymetrix, Santa Clara, CA, USA) according to the protocol provided by the manufacturer. Informatics analysis of the SNP array data was conducted using the CNAG/AsCNAR software, which enables the accurate detection of allelic status without paired normal DNA, even in the presence of up to 70–80% normal cell contamination^50^. Isochromosome 12p was confirmed when a sample had two more copies of 12p than of 12q. To confirm the copy number abnormalities detected using the SNP array, NGS-based copy number analyses were also performed in all samples using the in-house pipeline, CNACS^51^, which enable digital karyotyping.

### Targeted capture sequencing

The DNA isolated from 51 GCT samples was analyzed via targeted capture sequencing using a SureSelect XT Custom Kit (Agilent Technologies) according to the manufacturer’s protocol. Our custom bait library (U-Tokyo Onco-panel ver.1) was designed for mutation profiling of pediatric cancers, including 381 targeted genes and regions (Supplementary Data 12). Sequence alignment and detection of gene mutations and structural variations were performed using our in-house pipeline, Genomon v.2.5.3.

### Mutational analysis

Gene mutations identified using the Genomon pipeline were first filtered to exclude sequencing/mapping errors and mutations of unknown significance using the following criteria: (i) including only exonic and splicing regions; (ii) excluding known variants listed in the 1000 Genomes Project (Nov 2010 release), National Center for Biotechnology Information SNP database (build 131), or National Heart, Lung, and Blood Institute Exome Sequencing Project 5400; (iii) excluding read depths <50; (iv) excluding unidirectional reads; (v) including variants with *P* value <10^−60^ through Empirical Bayesian mutation Calling^52^; and (vi) including variants showing “H,” “M,” or “L” in the MutationAssessor. Moreover, mapping errors were removed via visual inspection of the Integrative Genomics Viewer browser.

### Reporting summary

Further information on research design is available in the Nature Research Reporting Summary linked to this article.

## Supplementary information

Supplementary Information

Description of Additional Supplementary Files

Supplementary Data 1–12

Reporting Summary

## Data Availability

All sequencing data supporting these findings, including DNA methylation arrays, RNA sequencing, targeted capture sequencing, and SNP arrays, were deposited in the DNA Data Bank of Japan and are accessible through accession number JGAS00000000204. All relevant data are available from the authors upon request.
