# Supplementary material for: Comprehensive genetic analysis of pediatric germ cell tumors identifies potential drug targets
Source: Commun Biol. 2020 Sep 30;3:544. doi: 10.1038/s42003-020-01267-8 (PMC7528104; doi:10.1038/s42003-020-01267-8)
Supplement: Supplementary file 1 — Supplementary Information [file 42003_2020_1267_MOESM1_ESM.pdf]

Supplementary Figure 1

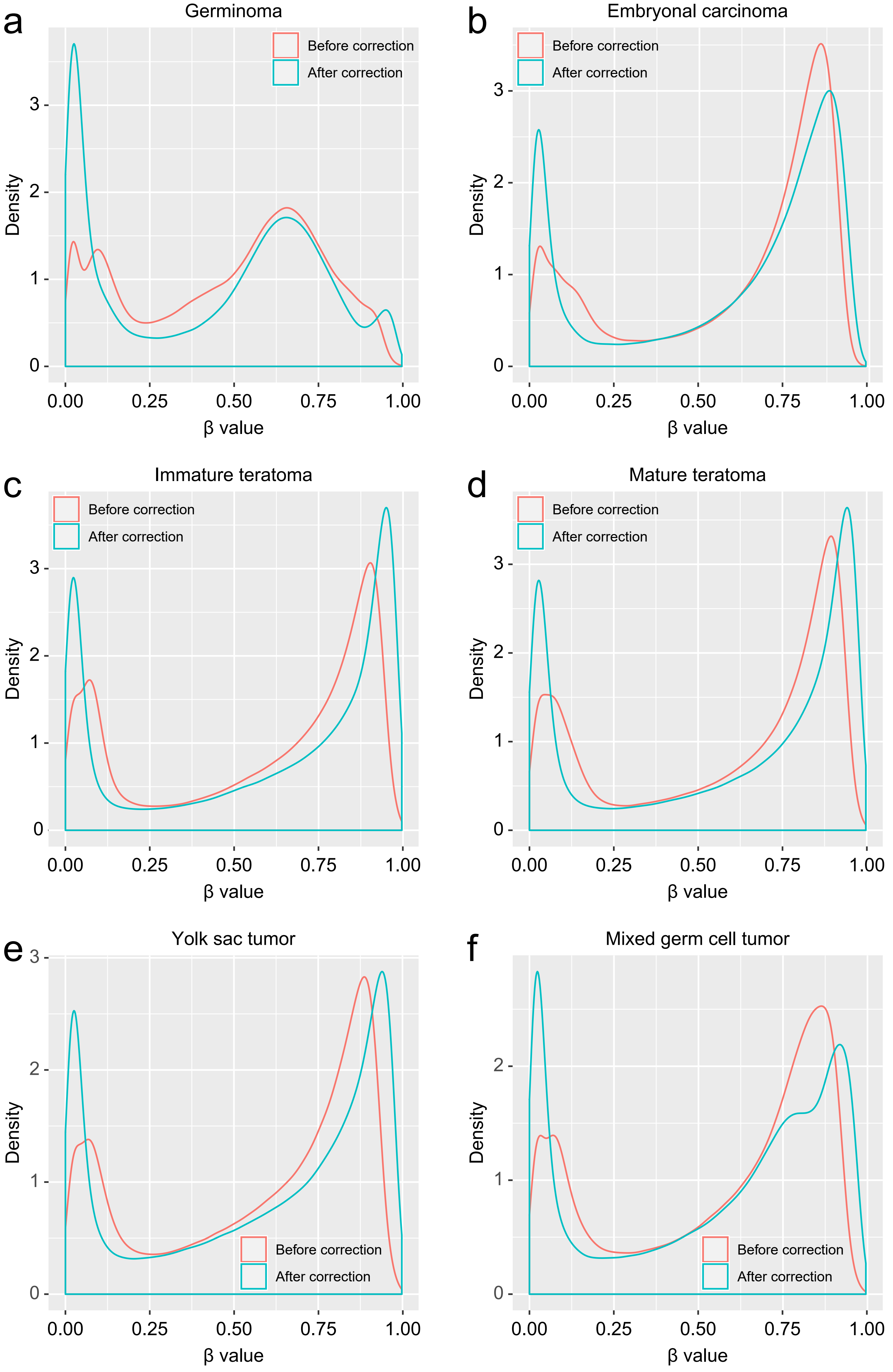

Supplementary Figure 1

DNA Methylation profile distribution of GEs (A), ECs (B), ITs (C), MTs (D), YSTs (E), and MGCTs (F).

The density plot describes the mean methylation profile of samples before correction (colored red) and after correction (colored green).

Supplementary Figure 2

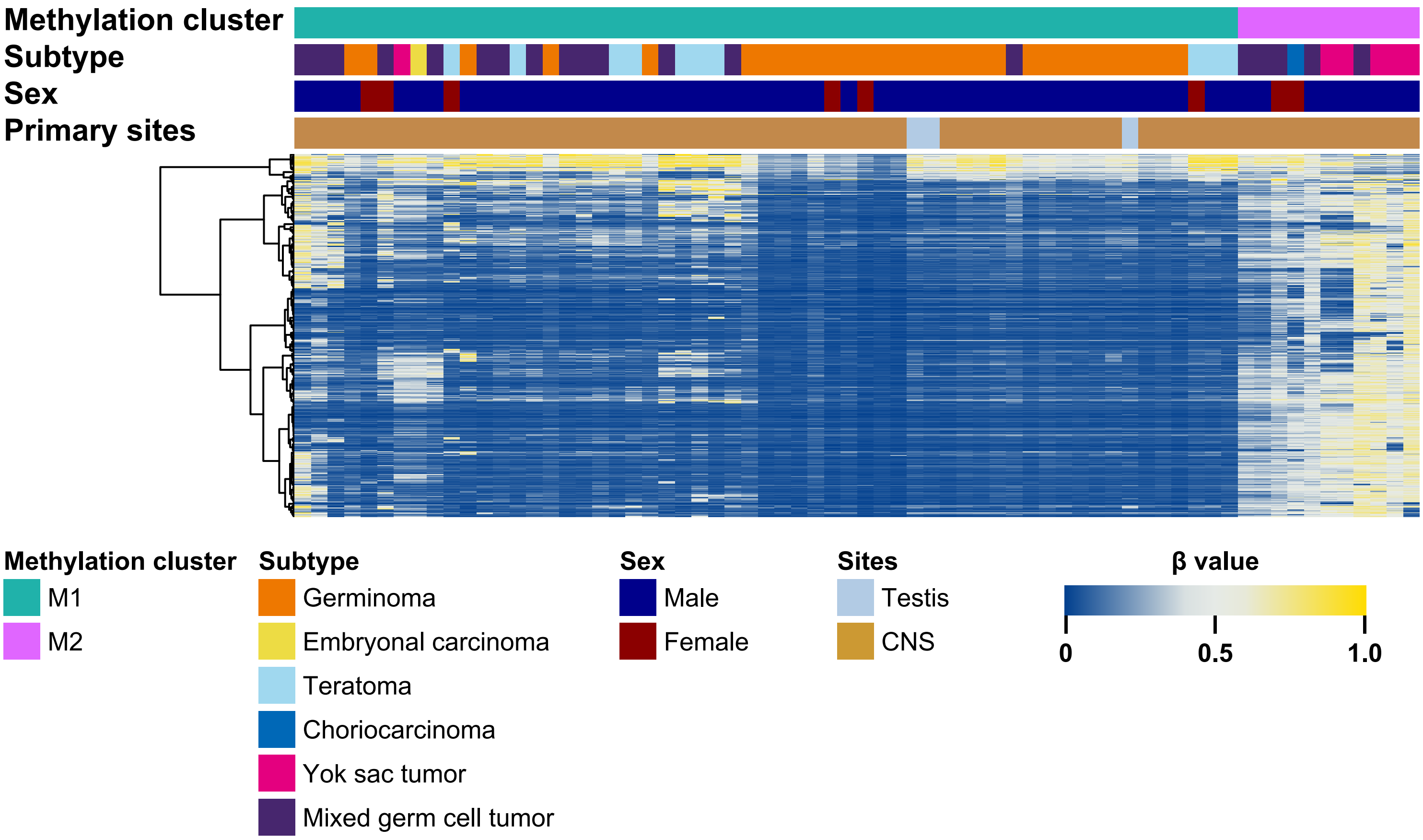

Supplementary Figure 2

DNA methylation clustering in 68 GCT samples on unsupervised consensus clustering. Consensus clustering revealed GCT samples were clustered into two clusters, M1 and M2. While M1 included several subtypes except YSTs, M2 included almost all of YSTs. DNA methylation clusters, subtypes, sex, and primary developing sites are shown by colors as indicated.

Supplementary Figure 3

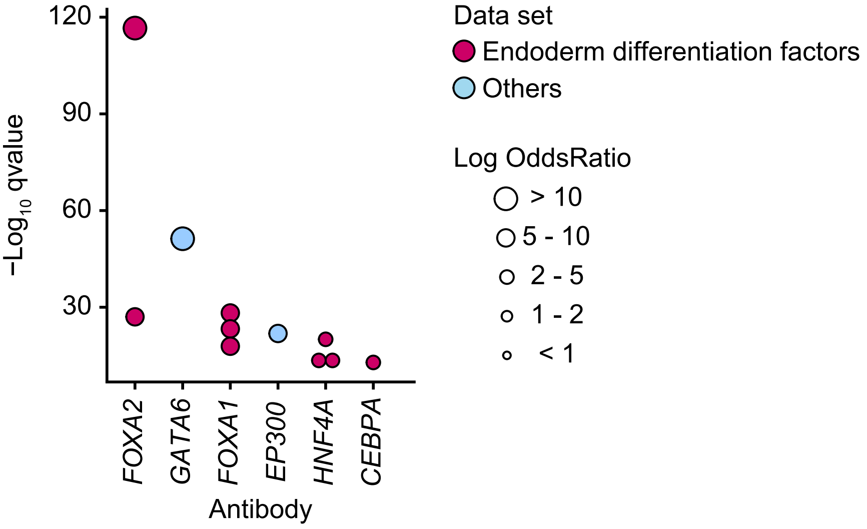

Supplementary Figure 3

The result of locus overlap analysis in the open data of YSTs. The binding sites of *FOXA2*, *GATA6*, *FOXA1*, *EP300*, *HNF4A*, and *CEBPA* were hypomethylated in YSTs. Colored dots represent ChIP-seq experiments for transcription factors. Dot size denotes the log-odds ratio.

Supplementary Figure 4

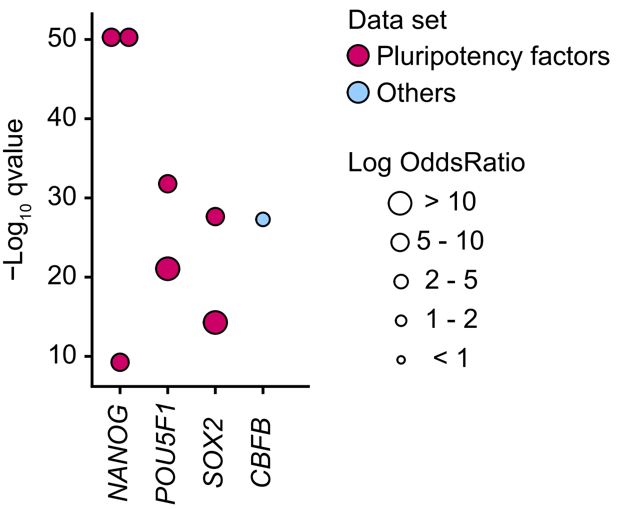

Supplementary Figure 4

The result of locus overlap analysis in the open data of ECs. The binding sites of *NANOG*, *POU5F1*, *SOX2*, and *CBFB* were hypomethylated in ECs. Colored dots represent ChIP-seq experiments for transcription factors. Dot size denotes the log-odds ratio.

Supplementary Figure 5

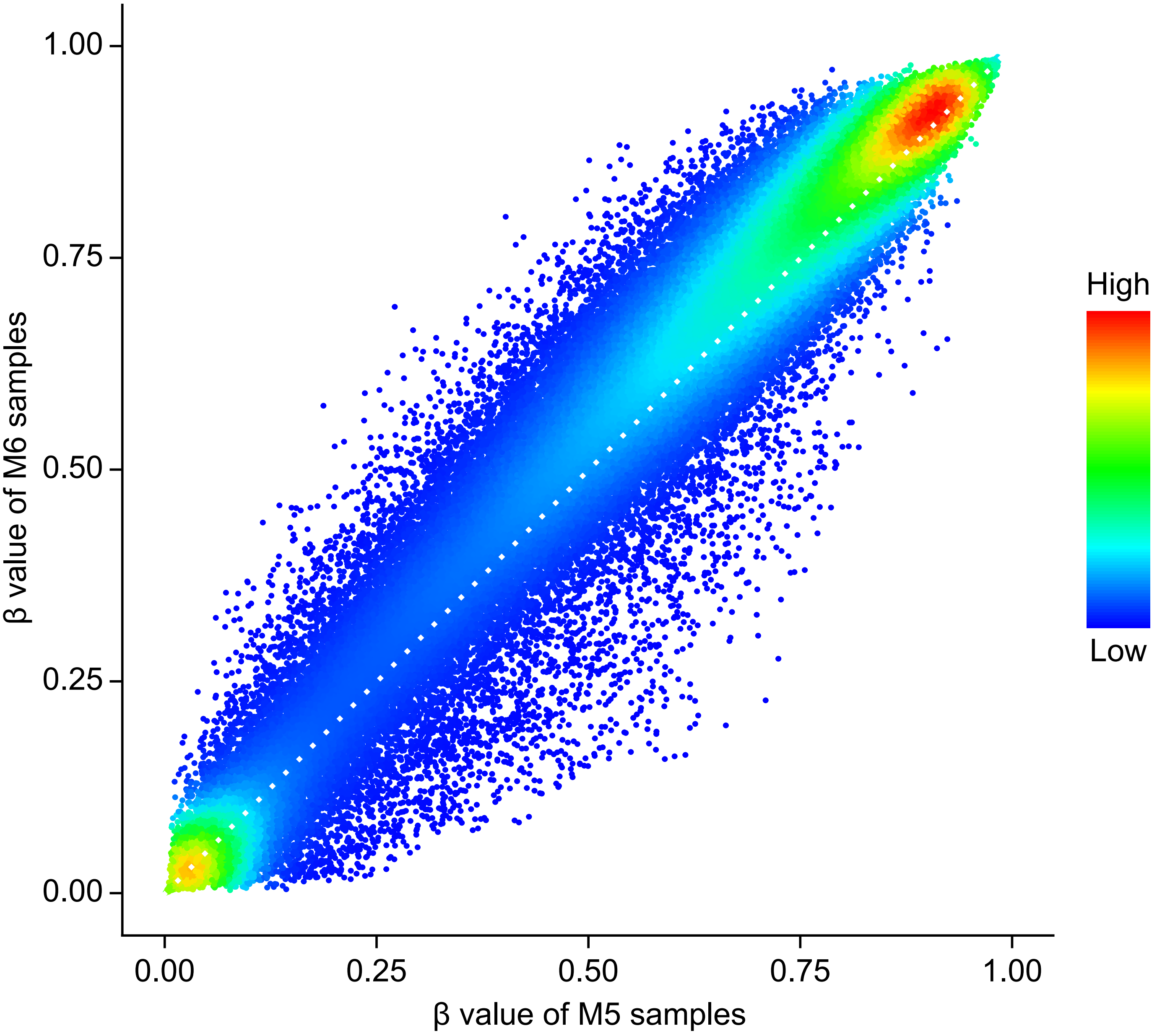

Supplementary Figure 5

Scatter plots and density color codes for DNA methylation data. The probes in M6 cluster samples were slight hypermethylated than those of M5.

Supplementary Figure 6

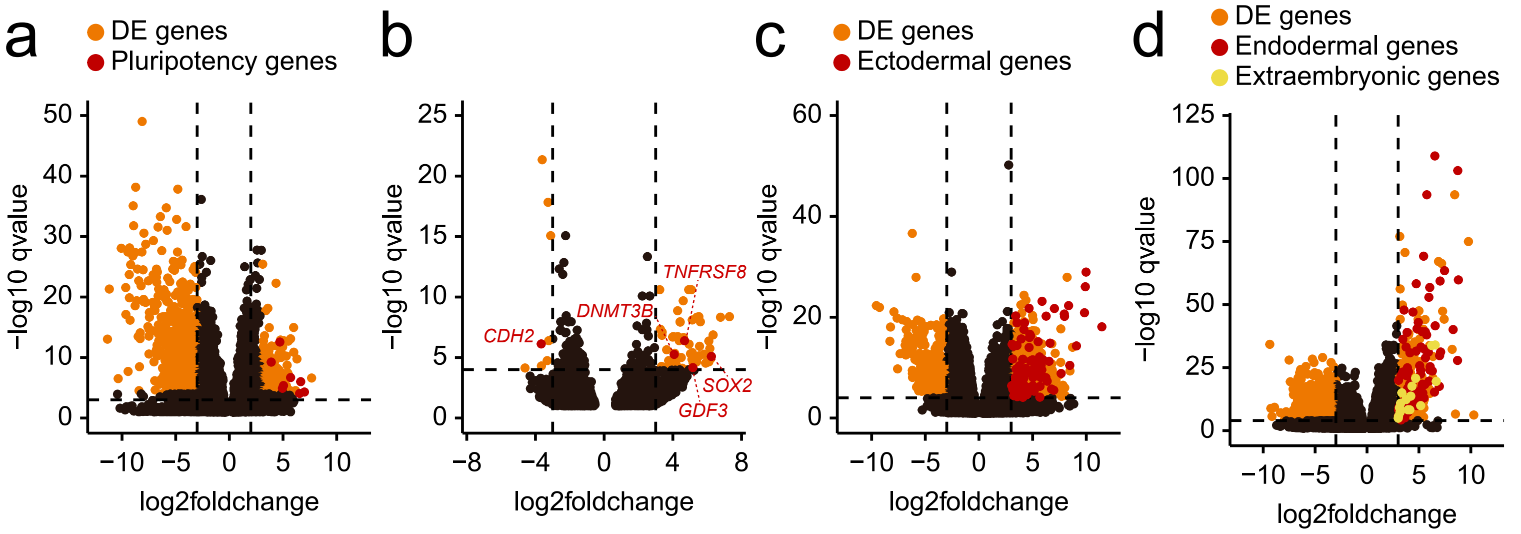

Supplementary Figure 6

Volcano plot comparing significant normalized expression counts between E1 and other clusters (A), ECs and germinomas (B), E2 and other clusters (C), and E3 and other clusters (D). Significant genes showing the log2 fold change of normalized expression counts greater than 3 or lesser than -3, and –log10 (q value) greater than 4 are colored in orange. Among these significant genes, pluripotency genes (A), ectodermal genes (C), and endodermal (D) genes are colored in red. Extraembryonic genes are colored in yellow (D). q values are calculated using the Wilcoxon rank-sum test following adjusted by Benjamini-Hochberg correction.

Supplementary Figure 7

Chromosome 12 of Sample GCT_033

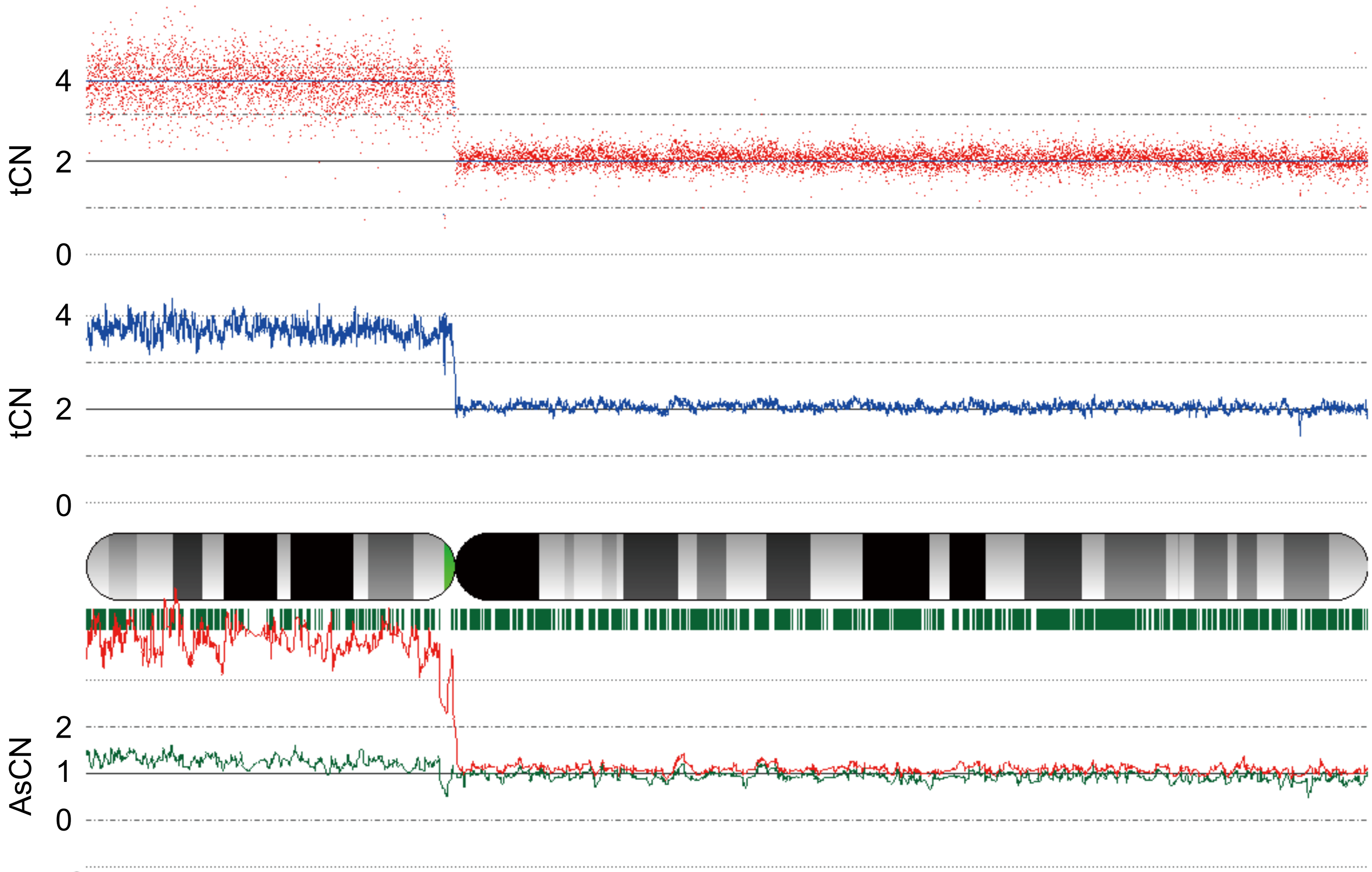

Supplementary Figure 7

Isochromosome of short arms of chromocome 12.

CNAG outputs for gains of short arms of chromosome 12. The moving averages of allele-specific copy numbers (AsCN) are depicted in red and green and the total copy number (tCN) are depicted in blue. The positions of heterozygous SNP calls (green bars) are also shown.

Supplementary Figure 8

Chromosome 1, 2, and 3 of Sample GCT_008

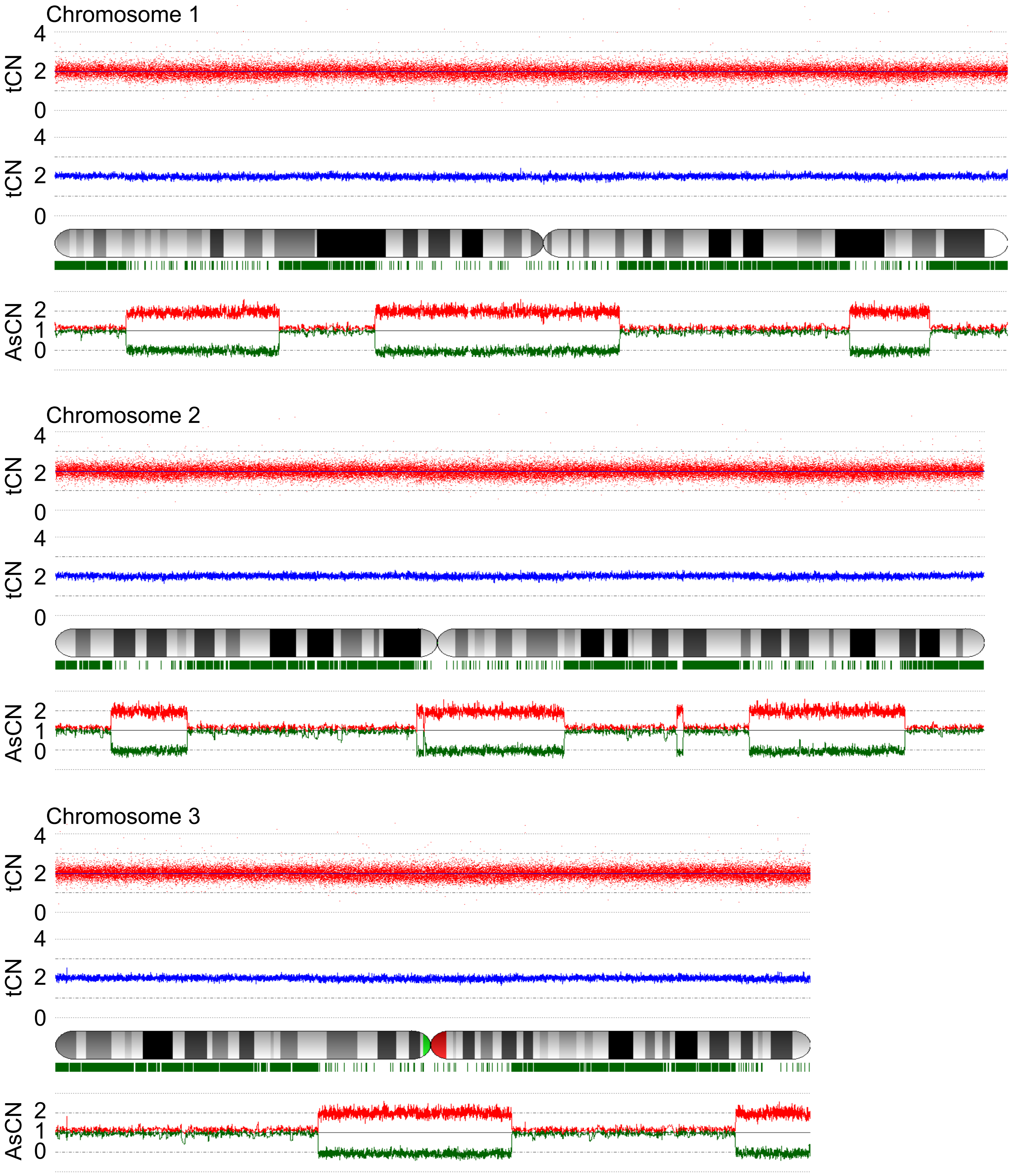

Supplementary Figure 8

CNAG outputs for uniparental disomies of chromosomes 1, 2, and 3. The moving averages of allele-specific copy numbers (AsCN) are depicted in red and green and the total copy number (tCN) are depicted in blue. The positions of heterozygous SNP calls (green bars) are also shown. Uniparental disomies, which are shown as splits in AsCN, are detected in each chromosome.

Supplementary Figure 9

Chromosome 1, 2, and 3 of Sample GCT_040

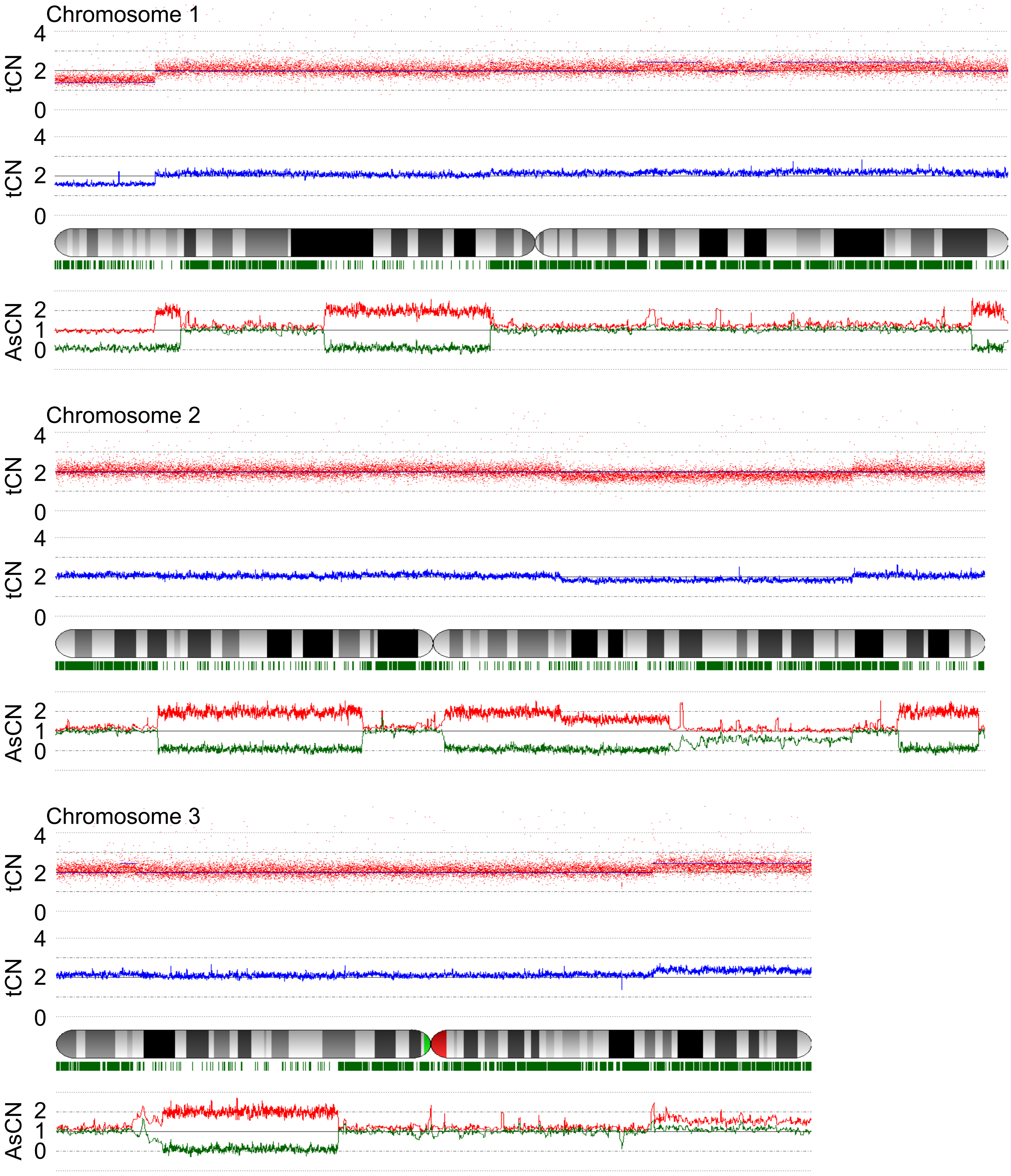

Supplementary Figure 9

CNAG outputs for copy number abnormalities of chromosomes 1, 2, and 3 in a yolk sac tumor sample. The moving averages of allele-specific copy numbers (AsCN) are depicted in red and green and the total copy number (tCN) are depicted in blue. The positions of heterozygous SNP calls (green bars) are also shown. In addition to uniparental disomies, copy number abnormalietis specific to yolk sac tumors such as the loss of chromosome 1q or gain of chromosome 3q are detected.

Supplementary Figure 10

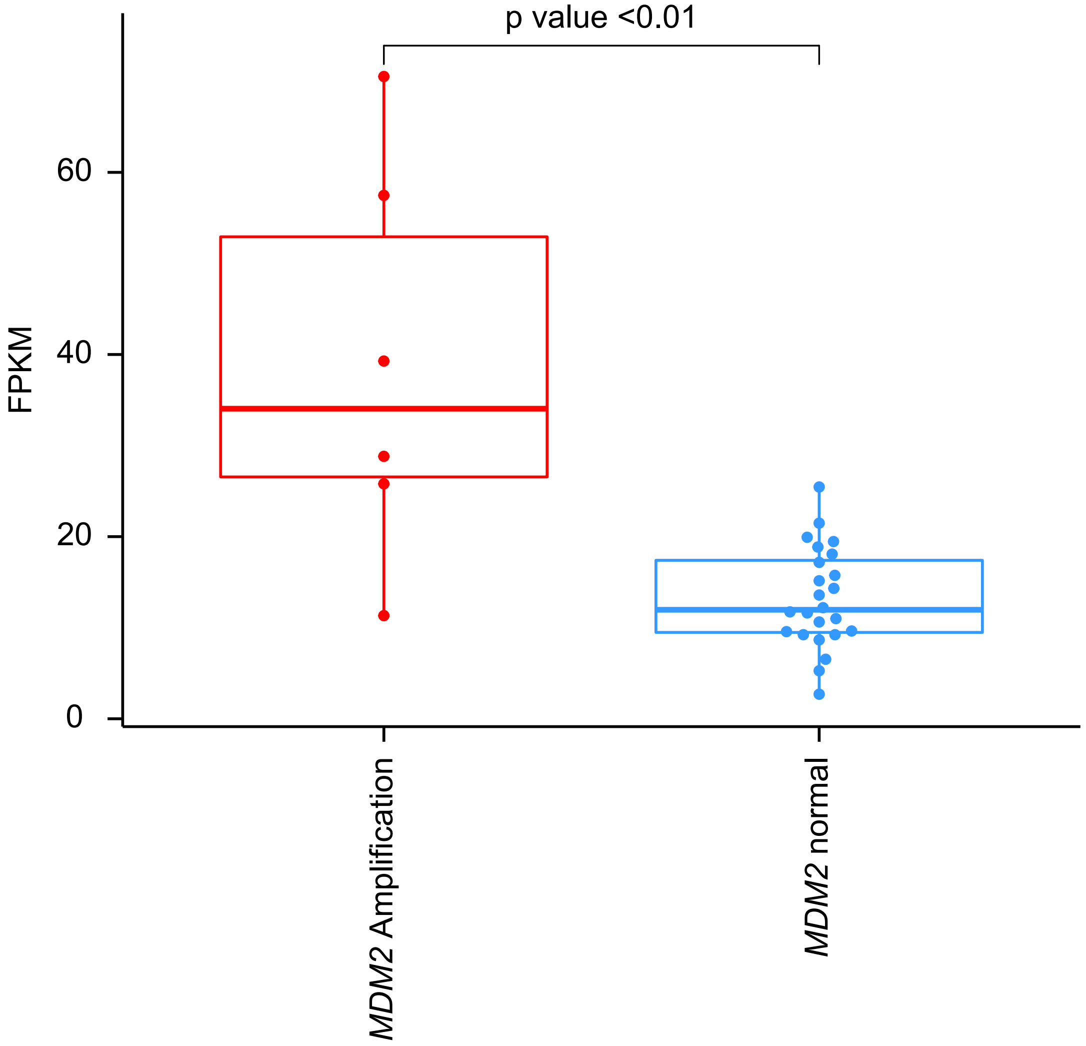

Supplementary Figure 10

Box plot comparing FPKM of *MDM2* expression levels in YSTs with *MDM2* amplifications (n=6) or not (n=24). In *MDM2*-amplified samples, *MDM2* were highly expressed than other samples. p value was calculated using the Wilcoxon rank-sum test.
