# Supplementary material for: Comprehensive genetic analysis of pediatric germ cell tumors identifies potential drug targets
Source: Commun Biol. 2020 Sep 30;3:544. doi: 10.1038/s42003-020-01267-8 (PMC7528104; doi:10.1038/s42003-020-01267-8)
Supplement: Supplementary file 2 — Description of Additional Supplementary Files [file 42003_2020_1267_MOESM2_ESM.pdf]

Description of additional supplementary items

Supplementary Data 1 – 12: This file contains the 12 Supplementary Data tables.

Supplementary Data 1: Patient characteristics of 51 GCT samples

Supplementary Data 2: Probes of first methylation clustering

Supplementary Data 3: DMPs between M1 and M2

Supplementary Data 4: LOLA results of M1vsM2

Supplementary Data 5: Probes of second methylation clustering in M1

Supplementary Data 6: Probes of second methylation clustering in M2

Supplementary Data 7: LOLA results of ECs

Supplementary Data 8: LOLA results of M5vsM6

Supplementary Data 9: Genes of expression clustering

Supplementary Data 10: KEGG upregulated pathways of E3

Supplementary Data 11: Mutations in GCT samples

Supplementary Data 12: Targeted genes and regions in U-Tokyo Onco-panel ver.1
